# New frontiers in porcine atrioventricular node decellularization: preserving extracellular matrix architecture for biological scaffolds

**DOI:** 10.3389/fbioe.2026.1766378

**Published:** 2026-03-13

**Authors:** Alice Tomas, Assunta Fabozzo, Domenico Ventrella, Nunzia Gallo, Alberto Elmi, Nicola Pradegan, Lucia Santorelli, Luisa Vera Muscatello, Tiziana Palmosi, Agnese Lauroja, Deborah Sandrin, Selena Mimmi, Ricardo Malvicini, Giada De Lazzari, Enrico Iaccino, Filippo Romanato, Giuseppe Sarli, Paolo Grumati, Luca Salvatore, Alessandro Sannino, Maria Laura Bacci, Anna Maria Tolomeo, Gino Gerosa

**Affiliations:** 1 Department of Cardiac, Thoracic and Vascular Science and Public Health, University of Padova, Padua, Italy; 2 Institute Pediatric Research ″Città della Speranza″, Padua, Italy; 3 Cardiac Surgery Unit, Hospital University of Padova, Padua, Italy; 4 Department of Veterinary Medical Sciences, University of Bologna, Bologna, Italy; 5 Department of Engineering for Innovation, University of Salento, Lecce, Italy; 6 Department of Veterinary Sciences, University of Pisa, Pisa, Italy; 7 Telethon Institute of Genetics and Medicine (TIGEM), Pozzuoli, Italy; 8 Department of Physics and Astronomy, University of Padova, Padua, Italy; 9 Department of Medical and Surgical Sciences, University “Magna Graecia”, Catanzaro, Italy; 10 Department of Women’s and Children’s Health, University of Padova, Padua, Italy; 11 Department of Experimental and Clinical Medicine, University “Magna Graecia”, Catanzaro, Italy; 12 Department of Clinical Medicine and Surgery, University of Napoli Federico II, Naples, Italy; 13 Typeone Biomaterials s.r.l., Calimera, Italy; 14 Department of Experimental Medicine, University of Salento, Lecce, Italy

**Keywords:** atrioventricular node, biological pacemaker, cardiac scaffold, decellularized extracellular matrix (dECM), tergitol-based protocol

## Abstract

**Introduction:**

Cardiac implantable electronic devices manage arrhythmias but are limited by mechanical failures, infection risks, and poor long-term biocompatibility. Developing a biological alternative that restores intrinsic pacemaking remains a key clinical challenge.

**Methods:**

We developed cardiac scaffolds from porcine atrioventricular nodes using an optimized Tergitol-based decellularization protocol. Morphological, ultrastructural, proteomic, and mechanical analyses were conducted to assess ECM integrity and preservation of native architecture.

**Results:**

The decellularization process effectively removed cellular and nuclear components while preserving three-dimensional structure, collagen content, and overall ECM organization. Analyses confirmed that key features essential for pacemaker tissue support were maintained.

**Discussion:**

Our findings demonstrate that the scaffold retains native characteristics suitable for biologically inspired pacemaker applications. This work provides a foundation for ECM-derived hydrogel development, cytocompatibility testing, and integration with cardiomyocytes in next-generation tissue-engineered cardiac scaffolds.

## 1 Introduction

The rhythmic contraction of the heart relies on the precise function of the cardiac conduction system, which coordinates the generation and propagation of electrical impulses throughout the myocardium (Bhattacharyya and Munshi, 2020). Disruption of this finely tuned network, whether due to congenital defects, degenerative changes, ischemic injury, or fibrosis, can lead to arrhythmias that severely impair cardiac function (Zakariyah et al., 2018; Theis et al., 2014; Nguyen et al., 2014). Conduction disorders, including sinoatrial and atrioventricular blocks, are among the most common rhythm disturbances and represent a major cause of morbidity (Barra et al., 2012). The increasing prevalence of cardiovascular disease and population aging has increased the number of patients requiring long-term rhythm management, underscoring the need for reliable and physiological therapeutic approaches.

Electronic pacemakers implantation remains the gold standard for treating conduction blocks and severe bradyarrhythmias (Mesirca et al., 2025). Although highly effective, these devices have intrinsic limitations. They provide non-physiological electrical stimulation that fails to fully mimic the natural electrophysiological behavior of cardiac pacemaker tissues. Moreover, device-related complications, such as lead fracture, infection, fibrosis, limited battery life, and repeated surgical replacement, pose significant clinical challenges. These limitations highlight the need for regenerative or biohybrid strategies that restore intrinsic excitability and conduction rather than merely substituting them artificially (Sriramoju et al., 2024).

In recent years, regenerative medicine and cardiac tissue engineering have opened new avenues toward biological alternatives to conventional pacemakers. Among these, scaffold-based approaches have shown promise, as they provide a three-dimensional microenvironment supporting cell attachment, maturation, and electromechanical coupling. Various synthetic and natural biomaterials, including poly (ε-caprolactone) (PCL), polyethylene glycol (PEG), poly (l-lactic acid) (PLLA) and poly (lactic-co-glycolic acid) (PLGA) or extracellular matrix (ECM) components (i.e., collagen, fibrin, hyaluronic acid), alginate, chitosan, silk proteins, and cellulose, have been explored for patch fabrication. Synthetic polymers offer tunable mechanical properties and processability but often exhibit poor hydrophilicity and slow degradation, sometimes releasing toxic by-products (McMahan et al., 2020; Khan et al., 2025; Satchanska et al., 2024). Conversely, natural polymers are typically more biocompatible and tissue-like but display limited mechanical strength and shorter lifespan. To address the limitations associated with synthetic and hybrid biomaterials, increasing attention has shifted toward decellularization-based strategies. These approaches enable the generation of biologically derived scaffolds that preserve the native extracellular matrix composition, ultrastructure, and three-dimensional organization, which are difficult to fully recapitulate using synthetic materials alone. The effectiveness of decellularization critically depends on the choice of detergent, as different agents exert distinct effects on cellular removal, ECM preservation, and residual cytotoxicity. Among available detergents, Tergitol, a non-ionic surfactant, has emerged as a viable alternative to Triton X-100. Community scientific researches and previously results obtained from our group showed Tergitol as an efficient detergent for decellularization while better preserving ECM architecture and biochemical cues in porcine tissues, including cardiovascular matrices (Mirosław et al., 2025; Palmosi et al., 2022; Tondato et al., 2023).

Despite significant progress, current cardiac patches still fall short of reproducing the distinctive ultrastructural, mechanical, and biochemical hallmarks of the sinoatrial node (SAN). Gluck et al. underscored the substantial disparities between engineered constructs and native pacemaker tissue, highlighting the challenge of mimicking its specialized microenvironment (G et al., 2017). In a different context, Lee et al. demonstrated a biohybrid swimming fish model powered by a cardiac-mimicking cell layer containing a self-activating pacing node, an elegant *in vitro* example of rhythm generation, yet still far from clinical translation (Lee et al., 2022).

Building upon this rationale, the present study aims to advance the development of biologically inspired scaffolds that closely replicate the pacemaking function of native conduction tissue. The overarching goal is to generate a next-generation, tissue-derived scaffold that could serve as a platform for creating implantable biological pacemakers. The porcine atrioventricular node (AVN) was selected as an experimental model due to its anatomical and electrophysiological relevance. Unlike conventional cardiac patches, which often lack the ultrastructural and biochemical complexity required for synchronized excitation, our approach focuses on preserving the native ECM architecture and protein composition essential for pacemaker activity. To this end, we optimized a decellularization protocol ensuring complete removal of cellular components while maintaining ECM integrity and three-dimensional organization. A comprehensive proteomic analysis was then performed to assess the retention of key structural and signaling proteins after decellularization. Finally, histological, imaging, and mechanical assessments were conducted to evaluate the scaffold’s morphological fidelity, compositional stability, and degradation profile.

## 2 Materials and methods

### 2.1 Atrioventricular node collection

Hearts were obtained from conventional hybrid pigs euthanized as controls in different experimental protocols approved according to the D. Lgs 26/2014, at the porcine experimental facility of the Department of Veterinary Medical Sciences of the University of Bologna (facility approval code 2216. A). Animals were euthanized by means of barbiturates overdose (thiopenthal sodium, 60 mg/kg; Pentothal Sodium, MSD), while under general anesthesia previously achieved by intramuscular injection of tiletamine/zolazepam (5 mg/kg; Zoletil, Virbac srl). Hearts’ weight ranged from 350 to 450 g. AVN were identified and isolated resulting in samples of approximately 1.5 cm^2^.

### 2.2 Tissue decellularization protocol

**FIGURE 1:**
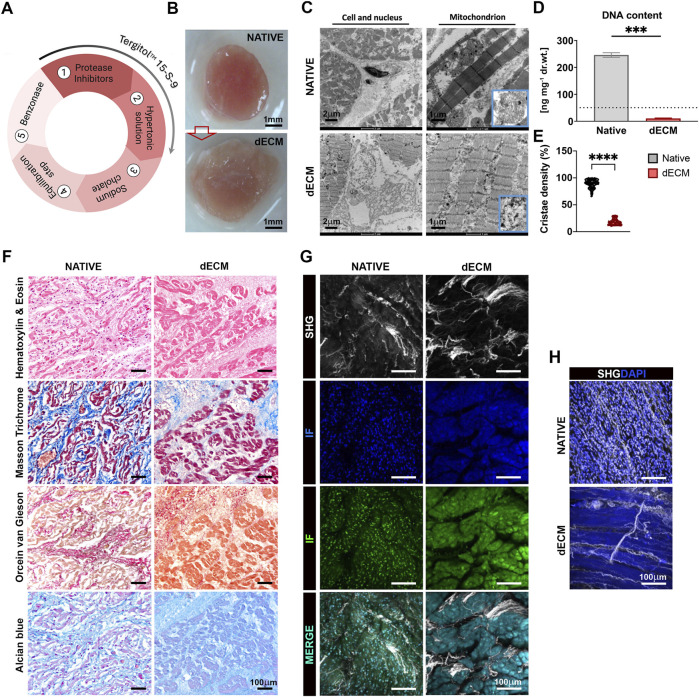
Decellularization efficiency of AVN tissue with Tergitol 15 S 9. **(A)** Decellularization process with Tergitol 15-S-9. The workflow includes an initial protease inhibitor treatment, followed by exposure to a hypertonic solution and subsequent sodium cholate-mediated decellularization. Intermediate washing steps were performed between these phases. An equilibration buffer step was then applied, followed by a final Benzonase treatment to remove residual nucleic acids. Tergitol was used during the protease inhibitor and hypertonic solution steps to support membrane solubilization. **(B)** AVN tissue biopsies of native and post-decellularization protocol (scale bar: 1 mm). **(C)** Representative microphotographs of native and decellularized cardiac tissues obtained by TEM respectively of cells (scale bar: 2 μm) and mitochondria (scale bar: 1 μm). Mitochondrial cristae close-ups in the blue boxes area are shown. **(D)** DNA quantification. The amount of DNA expressed as ng/mg dry tissue weight (n = 6 different pigs for native samples and n = 6 different pigs for decellularized samples) was significantly reduced, compared to the native (p-value <0.0001). **(E)** Analysis of mitochondria was carried out by quantifying the density of mitochondrial cristae. A total of 30 images per area were analyzed. Results were expressed as median ± range and significance were determined for independent samples using one-way ANOVA with *post hoc* Tukey’s test (n = 6 different pigs for native samples and n = 6 different pigs for decellularized samples). ****P < 0.0001. **(F)** Histology of the AVN. Native tissue: normal histological features of the AVN, characterized by nucleated nodal cardiomyocytes embedded in a collagen matrix, hematoxylin and eosin, 40× (scale bar: 100 μm); the interstitial collagen fibers are highlighted in blue, Masson’s trichrome, 40× (scale bar: 100 μm); elastic interstitial fibers are evident in black, Orcein Van Gieson, 40× (scale bar: 100 μm), and ECM light blue with Alcian 40x (scale bar: 100 μm). dECM tissue: the AVN cardiomyocytes show preserved shape and cytoplasmic borders and absent nuclei, hematoxylin and eosin, 40× (scale bar: 100 μm); interstitial collagen aggregation and dissolution in blue, Masson’s trichrome, 40× (scale bar: 100 μm); elastic fibers dissolved and fragmented, in black, Orcein Van Gieson, 40× (scale bar: 100 μm); ECM unchanged with respect to control, light blue, Alcian Blue, 40× (scale bar: 100 μm). **(G)** SHG imaging for collagen detection. The presence of cells in native tissue is visible even without DAPI due to their autofluorescence signals in the blue and green channels. Scale bar: 100 μm. **(H)** DAPI staining further highlights cellular nuclei. In contrast, the dECM sample showed strong autofluorescence indicative of matrix preservation and absence of cellular components. Scale bar: 100 μm.

AVNs were subjected to a multi-step decellularization protocol involving Tergitol 15-S-9. Tissues were rinsed with phosphate buffer saline (PBS) 1X (Sigma, cod. P3813) and consequently decellularized combining non-ionic detergent Tergitol 15-S-9 (Sigma, cod. 15S9) and sodium cholate hydrated (Sigma, cod. C1254) with the following procedure (Figure 1A). Briefly, samples were placed in sterile jars and treated with proteases inhibitors cocktail composed of 400 mM Phenylmethanesulfonyl fluoride (Thermofisher scientific, cod. 215740500), 1M N-Ethylmalemide (Merck, cod. 04260), 200 mM Iodoacetamide (Sigma, cod. I1149) and 1M Benzamidine (Sigma, cod. 12072), dissolved in DMSO for 16h with solution replacement after 8h. This step was followed by a wash in milliQ water for 8h. Subsequently, tissues were treated with a hypertonic solution composed of 0.5M NaCl (Sigma, cod. S9888) dissolved in PBS for 16h, with solution renewal after 8h, and then rinsed with milliQ water and Phosphate-Buffered Saline (PBS) for an additional 8h. 0.1% (v/v) Tergitol (Sigma, cod. 15S9) was included in all the aforementioned steps. A final treatment step with 10 mM sodium cholate dissolved in PBS was performed for 16h, with solution change after 8h. All solutions were prepared in a pH-buffered medium consisting of 0.1M sodium ascorbate (Sigma, cod. C1254) and 0.05M EDTA (Sigma, cod. E4884) in PBS which ensured 7.4 pH stability throughout the process. Each treatment step was conducted under constant agitation at 4 °C, except for the sodium cholate incubation, which was carried out at room temperature. After these steps, tissue samples were thoroughly washed with PBS 1X for 3h, with solution replacement after 1.5h. Then a 0.15 M NaCl solution was prepared by dissolving NaCl in a water:2-propanol (Carlo Erba, cod. 529091) mixture (9:1, v/v) and samples were incubated for 2h at 4 °C. Then samples were subjected to the equilibration step based on incubation with 50 mM Trizma® base (Sigma, cod. T1503), 0.5 mM Cl2 Mg (Sigma, cod. M8266) and milliQ water, pH 8, for 2h at 37 °C. Residual nucleic acids were digested using 1500 U/cm2 of Benzonase™ nuclease (Merk, cod. E1014), a non-specific endonuclease at 37 °C/ON. According to the protocol previously settled in our laboratory, samples were subjected to a high-level sterilization protocol adapted from previously published works (Palmosi et al., 2022; Fidalgo et al., 2018). Briefly, tissues were incubated in an antibiotic/antimycotic solution containing vancomycin, cefoxitin, gentamicin, ciprofloxacin, and amphotericin B at 37 °C for 24 h under agitation. Following treatment, samples were extensively washed with sterile phosphate-buffered saline (PBS) to ensure removal of antibiotic and antimycotic residues prior to further processing.

### 2.3 Histology and histochemistry

Decellularized and control samples were fixed in 10% formalin and trimmed according to the anatomical orientation of the AVN. Three-micrometer-thick sections were cut from paraffin-embedded tissue and routinely stained with Harris’ hematoxylin and eosin (H&E). Histochemical stains used were Masson’s Trichrome, Orcein-Van Gieson, and Alcian Blue. These were performed using the following commercially available kits, respectively: Masson Trichrome Kit (Bio-Optica S. p.A., cod. 04-010802); Elastica-Van Gieson Kit (Histoline Laboratories S. r.l., cod. wvgb-100T); and Alcian Blue Kit pH 2.5 (Bio-Optica S. p.A., cod. 04-160802). In each case, the staining procedure was carried out as recommended by the manufacturer.

### 2.4 DNA quantification

After the decellularization treatment, a quantification of DNA was performed to verify the efficacy of the process. DNA quantification was performed on AVN samples (n = 6 different pigs for native samples and n = 6 different pigs for decellularized samples) with a dry tissue weight ranging between 15 and 20 mg. DNA extraction was performed using the DNeasy® Blood & Tissue kit (Qiagen, cod. 69504) following manufacturer’s instructions. Subsequently, the concentration was measured using a NanoDrop 2000 spectrophotometer (ThermoFisher Scientific) at a wavelength of 260 nm. Final DNA measurements were reported in ng/mg tissue.

### 2.5 Scanning electron microscopy

To assess the influence of the decellularization treatment on the scaffold macro architecture, the morphology of the tissue surface was evaluated using a scanning electron microscope (SEM) (JEOL JSM-6490, Peabody, MA, United States) with particular attention to the dynamism of collagen, which is the main component of decellularized ECM (dECM). Both native and dECM node tissues were previously fixed in 8% (w/v) Karnovsky Paraformaldehyde (PFA) solution, 10% (v/v) glutaraldehyde (50%), and 40% (v/v) cacodylate buffer (0.2 M), pH range 7.2–7.4, at +4 °C in the dark; all reagents were supplied by Sigma Aldrich-MERK. Prior to analysis, tissues were rinsed in PBS and dehydrated through ascending solutions of ethanol (70%, 80%, 90%, 100%) for 10 min each. They were then vacuum dried in a critical point desiccator to replace ethanol with liquid CO_2_ and avoid tissue damage. Finally, the samples were coated with gold and palladium to create a conductive layer on the surfaces of the samples. The images were acquired in low vacuum mode at 20 kV, at different magnifications (n = 6 different pigs for native samples and n = 6 different pigs for decellularized samples).

### 2.6 Transmission electron microscopy

Small pieces of tissues (about 2–3 mm^3^) were fixed with 2.5% glutaraldehyde plus 2% paraformaldehyde in 0.1 M sodium cacodylate buffer pH 7.4 O/N at 4 °C. Subsequently samples were postfixed with 1% in 0.1 M sodium cacodylate buffer for 1 h at 4 °C. After three water washes, samples were dehydrated in a graded ethanol series (Carlo Erba, cod. 5291412) and embedded in an epoxy resin (Sigma-Aldrich). Ultrathin sections (60–70 nm) were obtained with an Ultrotome V (LKB) ultramicrotome, counterstained with uranyl acetate and lead citrate and viewed with a Tecnai G2 (FEI) transmission electron microscope (TEM) operating at 100 kV. Images were captured with a Veleta (Olympus Soft Imaging System) digital camera. Average thickness and spacing of collagen fibers were measured in TEM images of both native and decellularized samples using ImageJ Fiji software (version 2.16.0/1.54p).

Mitochondrial Analysis (%). Changes in mitochondrial cristae morphology were quantified by counting mitochondria with reduced lamellar density and/or abnormal lamellar structure, and by expressing these mitochondria relative to the total number of mitochondria counted. A total of 30 images per area were analyzed. Values are shown as median ± range (n = 6 different pigs for native samples and n = 6 different pigs for decellularized samples) (Tolomeo et al., 2024).

### 2.7 Mass spectrometry

All experiments were performed in a labelling-free setting. Biopsies from tissues were lysated in Radioimmunoprecipitation assay (RIPA) buffer implemented with protease inhibitors, specifically for each condition, three biological samples obtained from three different animals were analyzed (n = 3 for native samples and n = 3 for decellularized samples). Thirty micrograms of total protein lysate were precipitated with cold acetone and then reduced and alkylated in a solution of 6 M Guanidine-HCl, 5 mM *tris*(2-Carboxyethyl)phosphine (TCEP), and 55 mM chloroacetamide. Peptides were obtained by digesting proteins with LysC (WAKO) for 3 h at 37 °C and with the endopeptidase sequencing-grade Trypsin (Promega) overnight at 37 °C. Peptide mixtures were concentrated and desalted using the Stop and Go Extraction (STAGE) technique, then analyzed by liquid chromatography–tandem mass spectrometry (LC–MS/MS) using data-independent acquisition (DIA) (Rappsilber et al., 2003).

Analyses were performed on a NanoLC 1,200 system (Thermo Scientific) coupled via nano-electrospray ionization to a Q Exactive HF quadrupole-Orbitrap mass spectrometer (Thermo Scientific). Peptide separation was achieved on a home-packed 75 µm C18-AQ column (1.9 µm particles) with a 60-min gradient at 300 nL/min: 7%–32% solvent B over 45 min, 32%–45% in 5 min, 45%–95% in 3 min, followed by re-equilibration to 5% in 5 min. DIA mode employed 32 variable isolation windows spanning 300–1,650 m/z. MS1 scans were acquired at a resolution of 60,000 and MS2 at 30,000, both with an AGC target of 3 × 10^6^. Maximum injection times were 60 m (MS1) and 54 m (MS2), with normalized collision energies set to 25%, 27.5%, and 30%.

### 2.8 Proteomic data analysis

The MS raw data were processed in Spectronaut version 19.8 (Biognosys) using the library-free workflow.

Protein identification and quantification were performed with standard search settings: dynamic precursor and orbitrap fragment ion mass tolerances, cysteine carbamidomethylation as a fixed modification, and methionine oxidation plus protein N-terminal acetylation as variable modifications. Enzyme specificity was set to trypsin/Lys-C, permitting up to two missed cleavages, with a minimum peptide length of seven amino acids. Spectral matching was carried out against the UniProt *Sus scrofa* reference proteome (UP000008227).

Protein-level identifications were filtered to a 1% false discovery rate (FDR) before export and downstream analysis. Quantitative statistical evaluation was conducted in Perseus (version 1.6.2.3), with initial filtering steps removing contaminants, reverse database hits, and identifications based solely on modified peptides.

Label-free quantification (LFQ) intensity ratios were log2-transformed, organized into groups, and further filtered to retain proteins with at least three valid values in one group. Missing values were imputed from a normal distribution. Pairwise comparisons of protein abundances between experimental conditions were performed using Student’s t-test with permutation-based FDR correction, applying a significance threshold of p < 0.05. Proteins exhibiting a log2-fold change ±>1 were classified as differentially expressed. Gene Ontology (GO) enrichment analysis was performed on the dysregulated proteins, focusing on the cellular component (GOCC) category. Significant GOCC terms were determined using Fisher’s exact test with an FDR cutoff of 0.2. Matrisome specificity analysis was conducted using MatrisomeDB 2.0 as a reference. Specifically, the full list of identified proteins was compared against the core matrisome and matrisome-associated categories in the database to determine the ECM proteome composition. The mass spectrometry proteomics data have been deposited to the ProteomeXchange Consortium (Deutsch et al., 2020) via the PRIDE (Perez-Riverol et al., 2022) partner repository with the dataset identifier PXD073301.

### 2.9 Multiphoton microscopy

Second Harmonic Generation (SHG) imaging was performed on decellularized porcine patches in comparison with the porcine native tissue by using a custom developed multiphoton microscope (n = 6 different pigs for native samples and n = 6 different pigs for decellularized samples). Briefly, an incident wavelength of 800 nm was adopted to detect the collagen’s SHG signal at 400 nm on the photodetector (GaAsP PMT with 395/25 nm bandpass filter). The Z-stack images were acquired, at a fixed magnification through the Olympus ×25 water immersion objective with 1.05 numerical aperture (1,024 × 1,024 pixels), averaged over 80 consecutive frames, with a pixel dwell time of 0.14 μs and a pixel width of 0.8 μm. For quantitative measurements, the raw uncompressed images were analysed by using ImageJ software. Coherency (C), was calculated for collagen to verify the local dominant orientation of the images using OrientationJ, an ImageJ plugin. The estimated parameter was bounded between 0 and 1, indicating respectively the absence (isotropy) and the presence (anisotropy) of dominant orientation. Furthermore, to describe the texture of the tissue matrix (fibre organization and distribution), Fast Fourier Transformation (2D-FFT) was used. Briefly, the transform-based texture analysis techniques convert the image into a new form using the spatial frequency properties of the pixel intensity variations, allowing to extract textural characteristics from the image.

### 2.10 X-ray micro-computed tomography

Native and decellularized AVN three-dimensional organization was visualized by means of a Skyscan 1072 X-ray microtomography system (Bruker, Munich, Germany) on the central area on freeze-dried samples of 8 mm diameter. The operational parameters of 21 kV and 105 μA for the sealed X-ray tube were used to carry out two-dimensional image analysis with a resolution of 2.98 μm. A radiographic projection of the sample was repeatedly taken for each angle during the scanning (scanning rotation of 180°) while the sample was turned with a step rotation of 0.3° around the Z-axis (Gallo et al., 2023). No filters were applied. X-ray absorption radiographs were acquired through the specimen, and a slice reconstruction was performed by Bruker NRecon software (Version 1.6.9.8). Datasets were processed by Bruker CTan (Version 1.20.8.0) software, for a fine alignment with the reference axis system, and by Bruker DataViewer (Version 1.6.0.0) and CTVox (Version 3.3.1) software, for 3D analysis of samples microstructure based on the absorption coefficients of X-rays.

### 2.11 Mechanical characterization

The mechanical properties of native and decellularized AVN were investigated via uniaxial compression tests and cyclic compression tests using a ZwickLine universal testing machine (Zwick/Roell, Ulm, Germany) equipped with a 10 N load cell. Entire native and decellularized AVN samples of about 10 × 10 × 5 mm were equilibrated in PBS 1X at room temperature for 2 h before being subjected to cyclic compression test (Quarta et al., 2021). Their width and thickness were measured with a Dino-Lite digital microscope (AnMo Electronics Corporation, New Taipei City, Taiwan). Uniaxial unconfined compression tests were performed until the samples reached 80% of their initial height with a crosshead speed of 1 mm/min. To compare the time-dependent behavior, cyclic loading test consisting of 100 compression cycles with a compressive strain of 10% with a frequency of 1 Hz and a compressive speed of 20 mm/min were performed (Yang et al., 2019; Ruixin et al., 2016). These values were selected because they were in the natural strain range experienced by heart and used in bioreactors for mechanical stimulation (Majid et al., 2020). Each specimen was positioned at the center of the testing machine plate. After the application of a pre-load of 0.01 N, PBS 1X was added to keep samples hydrated for all the test time. The strain (e) was defined as (L_0_-L_n_)/L_0_, where L_n_ is the decreased sample thickness and L_0_ is the initial thickness. The stress (σ) was defined as F/A, where F is the compressive force and A is the cross-sectional area of the sample. Mechanical properties were calculated by means of Hooke’s law (Guimarães et al., 2020). The compressive modulus (E) and the maximum stress (σ_max_) were calculated by means of the software Microsoft Excel (Microsoft, WA, United States). In particular, E was calculated via linear regression of the linear region of the stress/strain curve while σ_max_ was calculated as the maximum stress value registered (Gallo et al., 2020; Monaco et al., 2017). For the cyclic loading tests integrals were carried out at hysteresis loop n. 1, 50, and 100 to record the amount of energy dissipation per volume unit of material (E_inc_). Cyclic compression tests were conducted on six independent specimens for each sample type. The viscoelastic properties of native and decellularized AVN were investigated by means of a Kinexus Pro parallel plate rheometer (NETZSCH Pumpen & Systeme GmbH, Waldkraiburg, Germany). A parallel plate geometry was chosen with an upper plate size of 25 mm diameter. Entire native and decellularized AVN samples (diameter: 25 mm, thickness: 0.5 mm) were cut to size using a scalpel, ensuring full coverage of the upper plate surface. Before being subjected to rheological analyses all samples were equilibrated in PBS 1X at room temperature for 2 h. The upper plate was slowly lowered to the selected gap under controlled conditions, allowing the samples to adhere homogeneously to the plate without inducing pre-stress. Prior to the test, samples were allowed to equilibrate at the testing gap for 5 min. Appropriated tests were performed to derive the dynamic or complex modulus, which is usually represented by the shear storage (G′) and loss (G″) moduli. The G′ represents the elastic, instantaneous and reversible response of the material while the G″ represents the viscous time dependent response of the material. In particular, the G′ and G″ moduli of native and decellularized AVN were recorded under a fixed frequency of 1 Hz over a shear strain range from 0.01% to 1,000% to determine both the linear viscoelastic range (LVR) and critical strain. Then, the G′ and G″ were recorded at specific strain from the LVR over a frequency range from 0.01 to 100 Hz. Finally, the viscosity of samples was recorded for shear rates from 0.001 to 1,000 1/s. All rheological analyses were performed at 37 °C. All sample types were tested in triplicate.

### 2.12 *In vitro* degradation resistance

The stability test was performed to assess the resistance to degradation of native and decellularized AVN samples under physiological-like conditions. Dry samples of about 1 cm × 1 cm were weighted and incubated in 5 mL of 0.1 mg/mL collagenase in PBS 1X at 37 °C (Gallo et al., 2021; Salvatore et al., 2020; Salvatore et al., 2021). For each prefixed time point, few microliters were withdrawn to determine the amount of protein degraded by means of the BCA assay kit (Merk, cod. 71285-M) (Smith et al., 1985; Brady and Macnaughtan, 2015).

### 2.13 ATR

FT-IR spectra were acquired using a FTIR-6300 from Jasco GmbH (Pfungstadt, Germany) in ATR mode on freeze-dried native and decellularized AVN samples. Absorption spectra were recorded in the range 4,000–600 cm^−1^ as an average of 64 scans at a resolution of 4 cm^−1^, normalized and smoothed according to the Savitsky–Golay method. Origin software from OriginLab Corporation (Northampton, MA, United States) was used for data analysis.

### 2.14 *In Vitro* angiogenesis assay

*Conditioned Medium Preparation*. Decellularized tissue samples from the AVN region (n = 6 different pigs), obtained under sterile conditions, were homogenized using an electric rotary homogenizer at 35,000 rpm (Cole-Parmer LabGEN 7) in Endothelial Cell Growth Medium-2 (EGM™-2) BulletKit™ (Lonza, cod. CC3162) (1 mg of treated tissue per 1 mL of medium). The medium was prepared following the manufacturer’s instructions as reported in the datasheet, which includes all recommended supplements and growth factors for HUVEC culture, such as fetal bovine serum, VEGF, bFGF, EGF, IGF, hydrocortisone, ascorbic acid, and heparin. Tissue homogenates were then incubated for 24 h at 37 °C in a humidified atmosphere with 5% CO_2_. This medium was referred to as “AV tissue homogenate medium (AV-THM).”

*Cell Culture and Angiogenesis Assay*. Human umbilical vein endothelial cells (HUVECs) were cultured in endothelial cell growth medium EGM™-2 Endothelial Cell Growth Medium-2 BulletKit™ (Lonza, cod. CC3162) under standard conditions and used between passages 3 and 6. For the angiogenesis assay, 96-well plates were pre-coated with Matrigel (Corning, cod. 354230) and incubated at 37 °C for 30 min to allow gelation. HUVECs were seeded at a density of [13,000 cells/cm^2^] and treated with either AV-THM, unconditioned control medium (healthy control), or control medium supplemented with DMSO (angiogenesis inhibition control). Cells were incubated for 24 h at 37 °C with 5% CO_2_.

*Image Acquisition and Quantitative Analysis*. At the end of the incubation period, images of the capillary-like networks were acquired using EVOS TM XL CORE PROMO microscope (MM Biotech). Tube formation was assessed using ImageJ software (NIH, United States) with the Angiogenesis Analyzer plugin. Quantitative parameters included the number of nodes, total tube length, and number of segments. All experiments were performed in triplicate unless otherwise stated.

### 2.15 Western blotting analysis

Native and decellularized tissue biopsies were homogenized in 1 mL of 1X RIPA buffer supplemented with complete protease inhibitor cocktail (Merk Millipore, Merck KGaA, Darmstadt, Germany) using the gentleMACS Dissociator and gentleMACS™ M Tubes (Miltenyi, Bergisch Gladbach, Germany) according to the manufacturer’s protocol (n = 6 different pigs for native samples and n = 6 different pigs for decellularized samples). The total protein concentration of each lysate was quantified using the Quick Start Bradford protein assay kit (Bio-Rad, Hercules, CA) with bovine serum albumin as the standard. Equal amounts (40 µg) of protein were separated by electrophoresis on NuPAGE® Novex®Bis-Tris Gels (Thermo Fisher Scientific, Waltham, MA) and transferred to nitrocellulose membrane. Membranes were blocked with 5% of not-fat dry milk in 1X Tris-buffered saline (TBS) containing 0,05% of Tween 20 and incubated with primary antibodies against proteins were detected using the antibodies anti-α-Gal (Enzo Life Science, cod. ALX-801-090), followed by the appropriate secondary antibodies. Specifically, goat anti-mouse IgG (H + L) (Thermo Fisher Scientific, cod. 62-6,520) and goat anti-rabbit IgG (H + L) (Thermo Fisher Scientific, cod. 31460), both diluted 1:4,000 in 5% milk in TBS containing Tween-20 (TTBS), were used. Protein bands were visualized using the Uvitec MINI6 imaging system (UVITEC, Cambridge, United Kingdom).

### 2.16 Statistical analysis

Statistical analyses were processed using GraphPad Prism 9 software, where a One-way Anova analysis was conducted with *post hoc* Tukey’s test for multiple comparison and Mann-Whitney U test to compare two groups, both using p < 0.05 as the threshold value for significance (p-value). *p < 0.05; **p < 0.01; ***p < 0.005; ****p < 0.0001. If not otherwise stated all data were reported as Mean ± SD.

## 3 Results

### 3.1 Efficient removal of cellular nuclei and DNA from the AV node matrix

AVNs decellularization involved several steps, from tissue inspection and sampling of the interest area to final decellularization (Figure 1A). A preliminary macroscopic analysis of the tissue showed a fainter coloration of the tissue and was less compact than the native one (Figure 1B).

The successful decellularization was confirmed by TEM imaging. As shown in Figure 1C, cells were clearly visible in the native tissue. In contrast, the decellularized tissue lacked cellular structures, leaving optically empty areas where they were previously located. To thoroughly investigate the degree of decellularization, the mitochondria status was also assessed. Figure 1C shows compact mitochondria with dense and well-defined mitochondrial cristae in the native tissue whereas, in the decellularized tissue, mitochondrial ultrastructure was disrupted, with completely disorganized mitochondrial cristae (89,25% ± 7,85% for native, 18,44% ± 5,54% for dECM) (Figure 1E). The amount of nucleic acids was below the threshold value (50 ng DNA/mg), confirming complete cell removal and, therefore, the effectiveness of the decellularization protocol (Crapo et al., 2011). Specifically, DNA content was 246.11 ± 24.30 ng DNA/mg in dry native tissue and 10.63 ± 3.97 ng DNA/mg in the dry decellularized tissue (Figure 1D).

Histological and histochemical images are shown in Figure 1F. Histological examination of the native sample revealed the presence of nucleated nodal cardiomyocytes embedded within a connective matrix rich in collagen fibers (stained blue with Masson’s trichrome), regularly arranged elastic fibers (visualized in black with Orcein van Gieson staining), and an alcianophilic ECM (appearing light blue with Alcian Blue staining). In contrast, decellularized samples showed preserved tissue morphology, with cell-shaped ECM structures suggesting maintenance of the native nodal architecture and an unchanged extracellular framework, indicating that the decellularization process effectively preserved the structural organization of the nodal matrix. In decellularized samples, the former cytoplasmic regions appeared with loss of cross-striations and absence of nuclei. The interstitial space showed mild dissolution of collagen fibers, faintly stained in blue with Masson’s trichrome, and fragmentation of elastic fibers, visualized as black strands and dots with Orcein van Gieson staining. Despite these changes, the overall ECM remained comparable to that of native tissue, with Alcian Blue staining suggesting apparent preservation of the general distribution of acidic polysaccharides and overall ECM organization. Label-free two-photon microscopy is a powerful imaging technique for evaluating scaffold architecture without the need for tissue staining or labeling, thus preserving sample integrity. To further confirm successful decellularization, autofluorescence analysis was performed. As shown in Figure 1G, autofluorescence emitted by nuclear components was detectable only in native tissue, while no nuclear signal was observed in decellularized samples, indicating complete removal of cellular material. To finally validate these findings, DAPI staining, a DNA-intercalating dye, was employed, confirming the absence of nuclei in decellularized tissue and supporting the autofluorescence results (Figure 1H).

### 3.2 Proteomic characterization of the native and decellularized AVN matrisome

**FIGURE 2:**
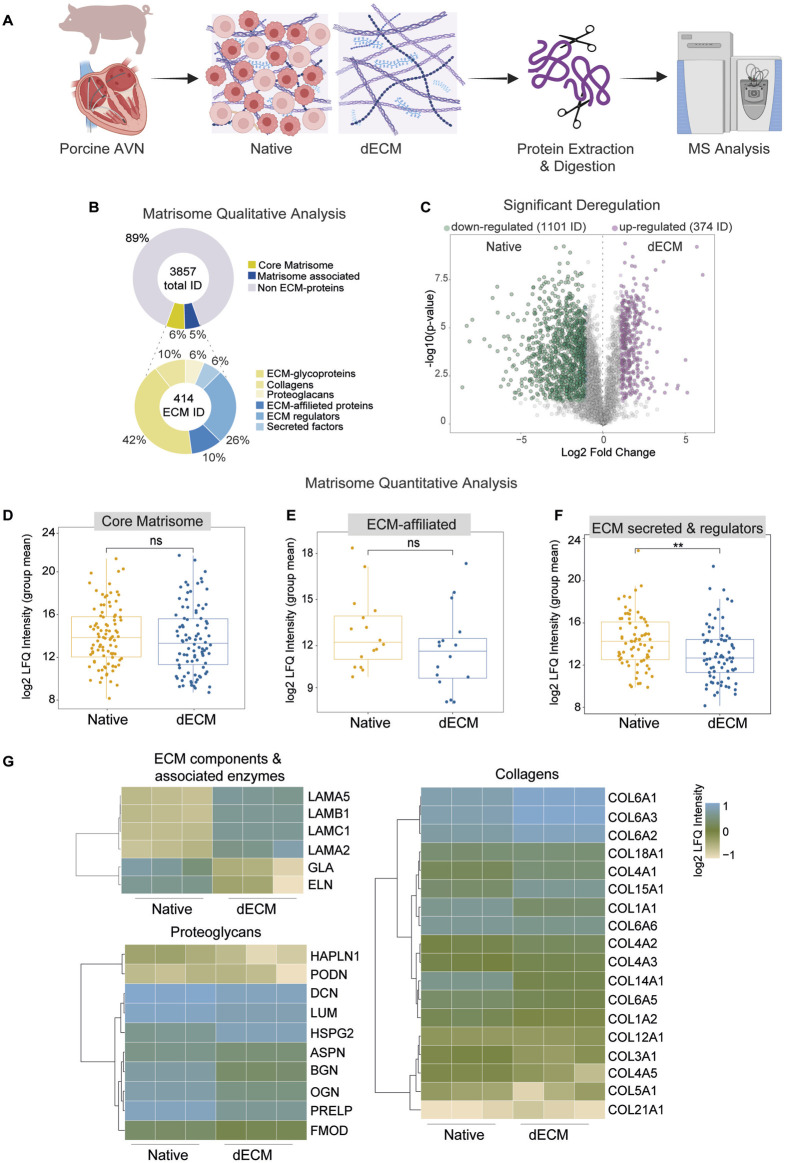
Proteomic comparison between native and decellularized AVN. **(A)** Diagram of the proteomic workflow applied. **(B)** Plots showing the distribution of all proteins identified in our dataset that matched MatrisomeDB 2.0. Proteins were first grouped into ECM main categories (upper) and then further subdivided into function-specific ECM subcategories (lower). **(C)** Volcano plot displaying differences in protein abundance levels between decellularized and native samples. **(D–F)** Box plot displaying the log2-transformed label-free quantification (LFQ) intensity (group mean) for core matrisome proteins **(D)**, ECM-affiliated proteins **(E)**, secreted ECM components and regulatory factors **(F)**, comparing decellularized and native conditions (ns = not significant; ** = p < 0.01). **(G)** Heatmaps showing the distribution of the group-mean LFQ intensities for selected proteins from the core matrisome and matrisome-associated categories, comparing decellularized and native conditions. (n = 3 different pigs for native samples and n = 3 different pigs for decellularized samples).

To assess the impact of the decellularization process on the ECM composition of the AVN, we performed a comprehensive proteomic characterization of native and decellularized samples (Figure 2A). Both qualitative and quantitative analyses were conducted to evaluate potential alterations in the matrisome. The qualitative profile identified that approximately 11% of total proteins belonged to the core matrisome and matrisome associated proteins. Among the matrisome-related proteins identified, glycoproteins, ECM regulatory proteins, collagens, and ECM-associated proteins represent the most abundant categories. Quantitative comparison between native and decellularized samples, performed using t-test analysis, revealed an overall decrease in total protein abundance in the dECM, consistent with the removal of cellular proteins during treatment (Figure 2B). Moreover, the comparison between native vs. decellularized tissues revealed an important downregulation of proteins in dECM tissues (1101 ID) that, however, presented an enrichment in 374 ID (Figure 2C). To further quantify differences in ECM composition, the core matrisome, ECM-associated proteins, and secreted and regulatory ECM proteins were compared between native and decellularized AVN samples. The analysis showed comparable expression levels for the central matrisome and ECM-associated protein categories between native and decellularized tissues, with no statistically significant changes (Figures 2D,E). Conversely, a significant difference was detected within the group of ECM-secreted and regulatory proteins, indicating a clear change in the abundance of these proteins following decellularization (Figure 2F). Heatmap analysis was performed to compare the relative abundance of ECM proteins between native and decellularized AVN samples. The visualization included proteins representative of the main ECM categories, namely, collagen, proteoglycans, ECM components, and associated enzymes. Collagen-related proteins (Col1A1, Col3A1, Col4A1) displayed comparable expression levels across both groups, suggesting maintenance of the main structural framework. Similarly, proteoglycans (decorin (DCN), biglycan (BGM), lumican (LUM), and perlecan (HSPG2)) and ECM-associated enzymes showed only minor variations without consistent directional changes. Altogether, the heatmap revealed a high degree of similarity between native and decellularized tissues, indicating that the overall distribution of proteins was largely preserved after the decellularization process (Figure 2G).

### 3.3 Preserved collagen structure with increased fiber directionality

**FIGURE 3:**
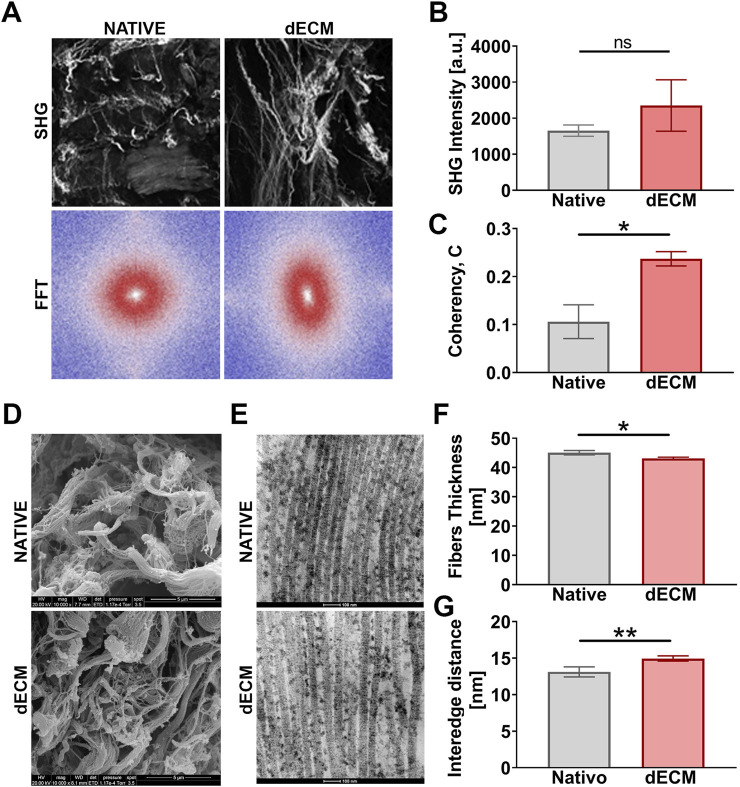
Ultrastructure analysis of collagen fibers after decellularization. **(A)** SHG of collagen fibers and 2D-FFT, from z-stack images, show fibers with different alignment. Scale bar: 100 μm. **(B)** Quantification of collagen fibers content with SHG intensity and **(C)** coherency of collagen which estimates the orientation of the fibers (n = 6 different pigs for native samples and n = 6 different pigs for decellularized samples). *p < 0.05. **(D)** SEM and **(E)** TEM of tissue to see collagen fibers preservation between native and decellularized (scale bar: 5μm and 100 μm respectively). **(F)** Fibers thickness and **(G)** interfibrillar distance evaluation. *p < 0.05; **p < 0.01.

Two-photon microscopy was employed to quantify collagen content and assess the preservation of fiber structure and orientation in both native and dECMs following the decellularization process. This technique enables the detection of SHG signals, which offer valuable insights into both the structural preservation of ECM proteins and the spatial organization of SHG-active fibrillar components, particularly collagen (Figure 3A). SHG intensity, which reflects the relative organization and density of fibrillar collagen, showed increased signal levels in decellularized tissue compared to native samples (Figure 3B). This apparent enrichment is likely attributable to the removal of cellular components, which increases the relative contribution of the collagen-rich extracellular matrix to the overall optical signal. (Borile et al., 2021). Specifically, the mean SHG intensity was 1655,37 ± 311,81 a. u. in the native group, compared to 2351,00 ± 1426,18 a. u. after treatment. Also, the degree of fiber alignment was quantitatively assessed through 2D-FFT analysis. This analysis revealed a shift in fiber orientation from an isotropic arrangement (i.e., spherical shape for FTT) in the native tissue to an anisotropic arrangement (i.e., ellipsoidal shape for FTT) in the decellularized tissue, with fibers tending to align along a single axis. This finding was further supported by the coherency calculation, showing an increase from 0.11 ± 0.07 in the native group to 0.24 ± 0.03 after decellularization (Figure 3C). Together, these imaging analyses provided a comprehensive overview of collagen organization, which was further examined at the ultrastructural level using SEM and TEM.

TEM and SEM were used to assess the preservation of the ultrastructure of the ECM, with focus on collagen and its fibrous organization, after the decellularization process. SEM analysis of the scaffold surface indicated that collagen fibers retained an organized micro-architectural arrangement, comparable to that observed in native tissue (Figure 3D). A slight thinning of fibers was visible, clearly ascribable to the decellularization process. Additionally, the preservation of collagen fibrillar ultrastructure was confirmed with TEM analysis (Figure 3E). Morphometric analysis performed on TEM images (Figures 3F,G) revealed an inverse trend between collagen fiber thickness and interfibrillar distance. Specifically, collagen fibers in the decellularized samples appeared thinner compared to those in native tissue (43,09 nm ± 2,39 for dECM, 45,06 nm ± 2,85 for native ECM), while the average interfibrillar spacing was greater (14,95 nm ± 1,88 for dECM, 13,12 nm ± 2,67 for native ECM), suggesting modest fibrillar reorganization without evidence of disruption of the underlying collagen framework following decellularization.

### 3.4 3D micro-computed tomography analysis revealed matrix compaction after decellularization

**FIGURE 4:**
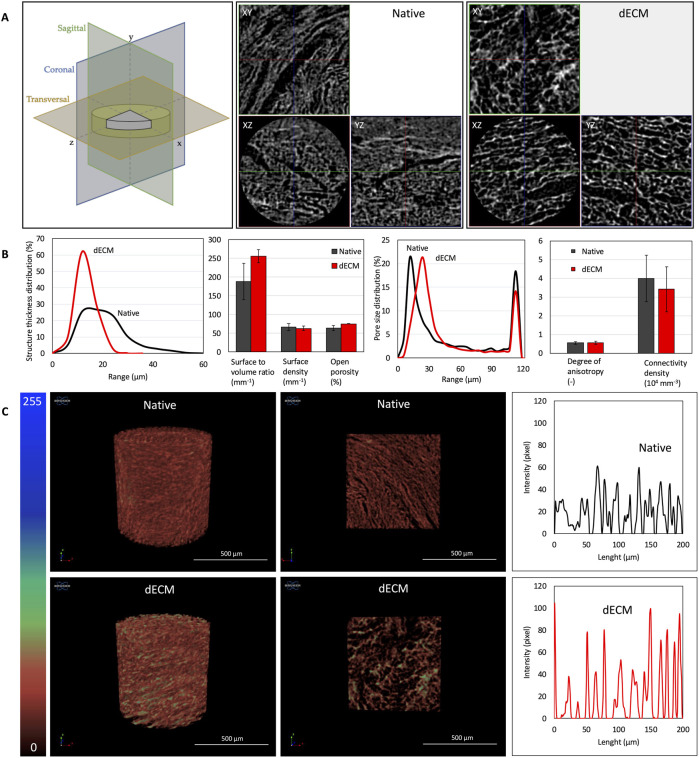
3D micro-CT analysis. **(A)** Representative coronal (Z‐Y, in blue), sagittal (X‐Y, in green), and transversal (X‐Z, in ocher) view of samples, where Z was the axis perpendicular to the native or decellularized AVN; **(B)** results from 3D analysis in terms of structure thickness distribution, pore size distribution, surface to volume ratio, surface density, open porosity, degree of anisotropy and connectivity density of native and decellularized AVN; **(C)** from left to right 3D reconstruction of μCT scans, their internal coronal slice in colored scale, and their relative intensity profile. Values reported represent the mean ± SD (n = 3 different pigs for native samples and n = 3 different pigs for decellularized samples).

The micro-Computed T omography (μCT) analysis was performed to assess native and decellularized AVN 3D organization. As shown in Figure 4A, the 3D structure of AVN was found to be largely homogeneous on the three-reference axis (x, y, z) and it was found to be affected by the decellularization process since a less bulky and denser structure was acquired. Indeed, sample pixel intensity profiles were found to be more intense (from 30 to 60 to 40-100 pixels) and separated (from 1-5 to 4–10 mm) (Figure 4C). Additionally, 3D analysis gave back an increase of the surface to volume ratio (189 ± 49 mm^-1^ for native ECM, 256 ± 16 mm^-1^ for dECM) and of the open porosity (64% ± 7% for native ECM, 75% ± 1% for dECM), that was ascribable to the removal of the cellular component (Figure 4B). Accordingly, a shift of the fibers thickness curve distribution peak to lower values and of pore size to higher values were found. Moreover, similar anisotropy degree (0.54 ± 0.07 for native ECM, 0.55 ± 0.09 for dECM) and connectivity density (3.99 ± 1.23 104 mm^-3^ for native ECM, 3.42 ± 1.20 104 mm^-3^ for dECM) were registered, suggesting the integrity of trabecular network upon the decellularization process.

### 3.5 Mechanical characterization

#### 3.5.1 Viscoelastic behavior and mechanical properties were preserved after decellularization

**FIGURE 5:**
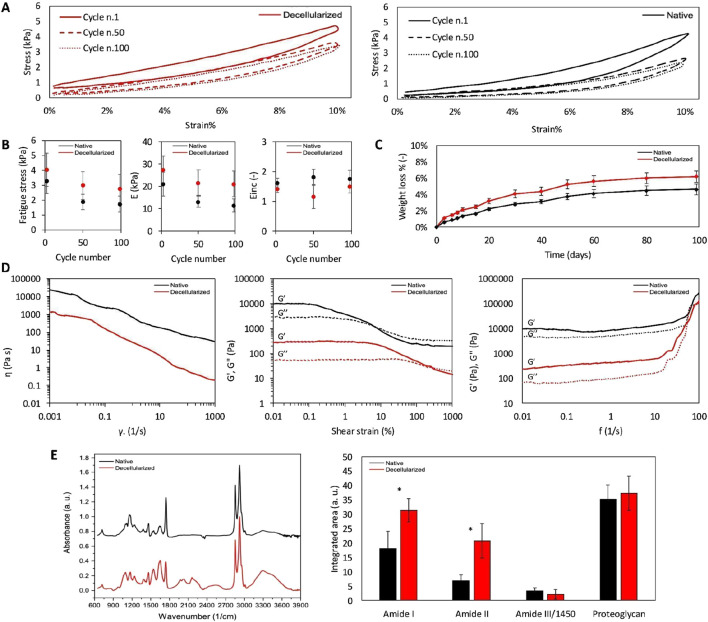
Mechanical characterization. Native (black) and decellularized (red) AVN representative hysteresis loops of loading cycles 1, 50 and 100 of cyclic compression test **(A)** with corresponding fatigue stress, stiffness **(E)** and the dissipated energy (Einc) values **(B)**; degradation resistance during time in simulated physiological conditions **(C)**; from left to right rheological properties investigated through viscosity vs. shear rate plot, G′ and G″ changes with respect to frequency at shear strains of 0.01%–1,000%, and G′ and G″ changes with respect to shear strain at frequencies of 0.01–100 Hz **(D)**; FTIR spectra of native and decellularized AVN recorded in the 4,000–400 cm^-1^ region **(E)**. Values reported represent mean ± SD (n = 6 different pigs for native samples and n = 6 different pigs for decellularized samples). *p < 0.05.

To comprehensively evaluate the functional behavior of native and decellularized AVN, both mechanical and rheological analyses were performed. Mechanical testing was conducted to assess the response of the materials to physiologically relevant compressive loads, providing insight into stiffness, fatigue resistance, and energy dissipation under repeated loading conditions. In parallel, rheological characterization was carried out to investigate the viscoelastic properties under shear deformation, which are critical for understanding tissue-like behavior, structural integrity, and potential performance under complex mechanical environments. The combined use of mechanical and rheological analyses enables a more complete description of the materials’ behavior, capturing both their load-bearing capacity and their time- and rate-dependent response, which are essential features for soft biological tissues and biomimetic matrices. First of all, native and dAVN mechanical properties were evaluated by cyclic compression tests to assess their strain reversibility capability under repeated loading. In Figure 5A, were representatively reported hysteresis loops number 1, 50 and 100 of the cyclic compression tests for both native and decellularized AVN. Both sample types exhibited well-defined hysteresis loops, with comparable profiles indicative of a nonlinear anisotropic viscoelastic constitutive behavior (Nordsletten et al., 2021). The fatigue stress, stiffness (E) and the dissipated energy (Einc) of each sample type was measured at the hysteresis loops number 1, 50 and 100 and reported in Figure 5B. Under compressive loadings, the decellularized AVN was found to be stiffer than native AVN. Although the initial stiffness values at cycle 1 were comparable between the two groups (p = 0.08), statistically significant differences emerged with increasing cycle number (pcycle100 = 0.004), primarily due to a progressive reduction in the mechanical properties of the native ECM. Specifically, the stiffness of decellularized AVN was not significantly affected by cyclic loadings (Ecycle1 = 27.6 ± 6.1 kPa; Ecycle100 = 21.2 ± 5.8 kPa; p > 0.05), whereas native AVN showed a marked decrease in stiffness over cycles (Ecycle1 = 21.0 ± 5.4 kPa; Ecycle100 = 11.5 ± 2.8 kPa; p = 0.003). A similar trend was observed for fatigue stress. Higher fatigue stresses were reached by decellularized AVN compared to native AVN across all compression cycles (p < 0.05), despite the comparable initial value at cycle 1 (p = 0.2). Additionally, while a significant reduction of fatigue stress was registered with increasing cycles number for native samples AVN (3.3 ± 0.8 kPa at cycle 1 to 1.7 ± 0.5 kPa at cycle 100, p = 0.004), no differences were revealed for decellularized AVN (4.0 ± 1.1 kPa at cycle 1, 2.8 ± 0.9 kPa at cycle 100, p = 0.06). On the contrary, Einc was comparable between native and decellularized AVN and did not significantly change between the first and last compression cycles (p > 0.05), indicating a similar energy dissipation capacity under cyclic loading.

Mechanical properties of materials are usually measured by means of Young’s modulus evaluation because of its independence from structure (Guimarães et al., 2020). However, in addition to uniaxial stress, biological tissues might also be subjected to deformations resulting from shear forces. Indeed, viscoelasticity is another important property to investigate, as the natural response of biological materials to stress is not purely elastic but also has a viscous component. As reported in Figure 5D (left panel), both native and decellularized AVN exhibited a non-Newtonian shear-thinning (pseudoplastic) behavior, with viscosity decreasing as the shear rate increased. Notably, decellularized AVN consistently displayed lower viscosity values across the entire shear-rate range, indicating a significant reduction in resistance to applied shear stresses following the decellularization process. Amplitude sweep results (Figure 5D, middle panel) further confirmed the viscoelastic nature of both native and decellularized AVN, with G′ and G″ values consisted with literature data (Biehl et al., 2022). Within the LVR, G′ predominated over G″ for both samples. Notably, G′ was found to be higher than G″ up to a shear strain of 10 ± 3% for native AVN, while for the dECM, the crossover point was found to be shifted to significantly higher shear strain values (82 ± 26%), indicating increased deformability. Frequency sweep analysis (Figure 5D, right panel), performed within the LVR range, further revealed that both matrices behaved as soft viscoelastic materials, with G′ exceeding G″. However, decellularized AVN showed consistently lower G′ and G″ values across the explored frequency range, indicating reduced stiffness and viscous dissipation capacity compared to the native tissue. Overall, mechanical and rheological data indicated that both native and decellularized AVN behave similarly to soft viscoelastic biological materials and stiffens under higher frequency loads. However, not negligible is the influence of the decellularization process on the matrix properties. In particular, it was found that decellularized AVN partially retained the characteristic viscoelastic response of soft biological tissues, exhibiting increased stiffness under compressive loading and a concomitant attenuation of its rheological properties under shear forces, ascribable to the cellular component removal (Sánchez-Cid et al., 2021).

#### 3.5.2 The fibrillar structure is well preserved following collagenase degradation assay

The degradation resistance of native and decellularized AVN was simulated *in vitro* and reported in Figure 5C. The weight loss observed was relatively small, with both native and decellularized samples showing less than 10% reduction in mass after 100 days, reaching a plateau in approximately 60 days. The dECM exhibited a slightly faster degradation rate compared to the native counterpart, a phenomenon that can be explained by the gentle removal of cellular components and part of the soluble proteins during the decellularization process, which increases matrix porosity and therefore susceptibility to hydrolytic cleavage. Nonetheless, the marginal difference between native and decellularized tissues suggests that the protocol employed preserved most of the fibrillar structure of AVN.

#### 3.5.3 Spectroscopic evaluation confirms preservation of ECM biochemical composition

The ATR spectra of native and decellularized AVN were reported in Figure 5E. As known, AVN is mainly composed of collagen and glycosaminoglycans (Efraim et al., 2019). Type I collagen is the most abundant followed by type III and others in traces (collagen type II, IV, V, VI, VII, VIII, XI) (Efraim et al., 2019). The presence of the collagenous component in both samples was revealed and confirmed by the detection of the typical peaks of the predominant type I collagen. In particular, amide I, amide II, amide III, amide A and B were revealed (Gallo et al., 2021; Prontera et al., 2023; Salvatore et al., 2024; De Campos Vidal and Mello, 2011; Carbonaro and Nucara, 2010; Singh et al., 2022). The amide I (1,621–1,635 cm^−1^) band, associated with C=O hydrogen-bonded stretching, was found at 1,641 cm^−1^ for the native and at 1,647 cm^−1^ for the decellularized AVN. The amide II (1,535–1,548 cm^−1^) peak, associated with C-N stretching and N-H in-plane bending from amide linkages, was found at 1,546 cm^−1^ for the native and at 1,540 cm^−1^ for the decellularized AVN. The amide III (1,220–1,240 cm^−1^), associated with the N-H bending, was found at 1,240 cm^−1^ for both native and decellularized AVN. The amide A and the amide B, associated to the N-H stretching coupled with intramolecular H-bond and N-H bend, were respectively found at 3,297 cm^−1^ and 3,201 cm^−1^ for the native and 3,292 cm^−1^ and 3,201 cm^−1^ for the decellularized AVN. Peaks attributable to the other collagenous species were also found. In addition to the peak at 1,464 cm^−1^, common to all collagen types, distinct peaks were found corresponding to specific collagens: 1,370–1,380 cm^−1^ (collagen I and IV), 1,160–1,170 cm^−1^ (collagen IV and V), and 1,081–1,100 cm^−1^ (collagen V and VI). Additionally, other contributions were found. In particular, the presence of glycosaminoglycans was confirmed by the peaks in the range 1,250–1,000 cm^−1^ attributable to C-O-C and C-OH bonds of carbohydrates. The characteristic absorption band of lipids was observed at about 1740 cm^−1^, which corresponds mainly to the ester carbonyl group and–CH groups (Huang, 2021). Being attributed to the COOH stretching vibration, the contribution at about 1740 cm^−1^ could be due to the pronounced collagen amount since it could originate from the protein backbone (Vásquez-Rivera et al., 2019). The asymmetric and symmetric -CH2 and -CH3 stretching bands from elastin and collagen structures were located at 2950–2850 cm^−1^ (Singh et al., 2022; Huang, 2021). Thus, the slight differences in peak presence and position revealed among native and decellularized AVN suggested that the decellularization process did not affect the general composition of the AVN ECM. However, from the evaluation and comparison of band area intensities, differences emerged. In particular, regarding the collagen content, decellularized samples presented a variation of about 50% for amide I and 70% for amide II. According to Almeida et al., this variation is not ascribable to an increase of the collagen content but to cell components removal that enhanced the spectral intensity of collagen molecular components (Almeida et al., 2023). No statistically significant differences in the proteoglycan content and in the collagen triple helical structure content (Amide III/1,450 cm^−1^ ratio) between native and decellularized samples were revealed.

## 4 Discussion

The development of biological alternatives to electronic cardiac pacing remains a key challenge in cardiac regenerative medicine, particularly due to the difficulty of reproducing the structural, biochemical, and mechanical features of native conduction tissue. In this study, porcine AVNs were decellularized using a Tergitol-based protocol, and the resulting scaffolds were extensively characterized to assess ECM preservation, microstructural organization, mechanical properties, and immunocompatibility.

Our results demonstrate that the Tergitol protocol effectively removes cellular components, as confirmed by histology, DNA quantification, TEM, and epifluorescence imaging. The AVN is a highly specialized cardiac structure with low cellular density and high ECM content; accordingly, native tissue DNA levels were lower than those reported for atrial or ventricular myocardium. Decellularization reduced DNA content well below the accepted threshold (<50 ng DNA/mg), with mitochondrial and nuclear structures largely absent, consistent with previous reports (Palmosi et al., 2022; Crapo et al., 2011).

Proteomic analyses showed that core structural components of the ECM, including major fibrillar collagens (Col1A2, Col3A1, Col4A1) and proteoglycans, were largely preserved following decellularization, supporting maintenance of the AVN. Quantitative differences mainly involved a selective reduction of regulatory and more soluble ECM-associated proteins. This pattern is consistent with their intra- or extracellular localization and higher solubility, which make these components more susceptible to extraction during detergent-based decellularization protocols. Importantly, this depletion reflects effective removal of cell-associated and labile proteins rather than disruption of the collagenous backbone as previously reported in proteomic studies of decellularized cardiac matrices (Krawczyk-Ożóg et al., 2025; Thorsell et al., 2024). Overall, the observed proteomic profile reflects the intrinsic trade-off of detergent-based decellularization, in which preservation of structural ECM components is accompanied by partial loss of more labile components. In particular, while major fibrillar collagens and proteoglycans were retained, matricellular proteins and soluble growth factors involved in cell signaling, tissue remodeling and paracrine regulation, were partially depleted, consistent with their higher solubility and closer association with cellular compartments. (Crapo et al., 2011; Gilbert et al., 2006). Although this loss does not compromise the structural integrity of the ECM, it may influence subsequent cell-matrix interactions and functional integration during recellularization. In this context, the use of Tergitol offers specific advantages. Compared with commonly used ionic detergents such as SDS, which achieve efficient cell removal at the expense of extensive ECM disruption, and classical non-ionic detergents such as Triton X-100, which may preserve fibrillar architecture less consistently, the Tergitol-based protocol provides a milder non-ionic approach that favors preservation of collagen fibrillar ultrastructure while sufficiently reducing immunogenic material, including α-Gal epitopes. (Palmosi et al., 2022; Fidalgo et al., 2018; Crapo et al., 2011; Gilbert et al., 2006; Keane et al., 2015). This balance is particularly relevant for specialized tissues such as the AVN, where ECM organization contributes to both mechanical support and coordinated electrical conduction. Nevertheless, this approach has limitations, including the potential loss of soluble regulatory ECM proteins and subtle microstructural alterations. SHG imaging and 2D-FFT analyses revealed increased collagen fiber alignment and reduced angular dispersion in decellularized samples compared to native tissue, without major changes in fibril morphology or thickness, as confirmed by TEM and SEM. These findings are consistent with previous reports on decellularized cardiac and vascular matrices (Marcos-Garcés et al., 2022; Barbon et al., 2022). In addition, decellularized scaffolds exhibited greater spatial variability in SHG intensity, indicative of a less homogeneous collagen network and local differences in fiber density and organization introduced during decellularization.

Mechanical testing further supported these observations, showing that decellularized AVNs retained elasticity, fatigue durability, and predominantly elastic behavior within their linear viscoelastic range. Under cycling loadings, native AVN samples exhibited a progressive reduction in fatigue stress with increasing cycle number, which can be attributed to the viscoelastic relaxation phenomena and microstructural rearrangement of the ECM components under repeated compressive loading. In native tissue, reorientation of cells, collagen fibers and proteoglycans along the load direction, together with their sliding and bulking promoted stress redistribution and energy dissipation. In contrast, decellularized AVN displayed relatively stable fatigue stress values across cycles indicating reduced extent of viscoelastic relaxation and structural rearrangement. This behavior is consistent with the removal of cellular components and the resulting dominance of a more mechanically stable, predominantly elastic fibrous network. In rheological analyses, decellularized AVN displayed markedly lower viscosity values over the entire shear-rate range, confirming a significant decellularization-induced reduction in resistance to applied shear rates. The broader linear viscoelastic range observed in decellularized samples is likely associated with increased collagen fiber alignment, in agreement with studies showing that collagen anisotropy modulates mechanical load distribution and cellular responses in cardiac scaffolds. (Nordsletten et al., 2021; Childers et al., 2021; Basara et al., 2021; Zhang et al., 2016). Thus, decellularized AVNs were found to exhibit increased stiffness under compressive loading and a concomitant attenuation of their rheological properties under shear forces, ascribable to the cellular component removal. This phenomenon can be explained by the load-mode dependence of AVN and the microstructural changes induced by decellularization. Removal of cellular components reduces viscous dissipation mechanisms and disrupts cell–matrix interactions that primarily contribute to shear resistance, leading to decreased viscosity and lower G′ and G″ values. Conversely, preservation and possible compaction of the fibrous ECM network following decellularization may enhance fiber–fiber interactions and load transfer efficiency under compressive deformation, resulting in increased compressive stiffness. From a physiological perspective, this distinction is particularly relevant, as AVN tissue *in vivo* is predominantly exposed to shear and tensile forces rather than purely compressive loads. Therefore, while decellularization improves mechanical stability under compression, it may concurrently reduce matrix resistance to shear, highlighting the importance of load-mode–specific mechanical characterization when evaluating decellularized matrices for physiological relevance or regenerative applications. Immunocompatibility analyses demonstrated effective removal of α-Gal epitopes and absence of cytotoxic or inflammatory responses in HUVECs cultured with decellularized AVN-conditioned medium. While these *in vitro* results are encouraging, *in vivo* immune responses are inherently, more complex, involving interactions with scaffold architecture, degradation products, and mechanical integration (Keane et al., 2015). Indirect biological assays, including angiogenesis tests (Supplementary Figure S1), were therefore performed as a preliminary safety assessments to screen for potential cytotoxic effects and should not be interpreted as preliminary safety evaluations rather than functional validation of cardiac-specific activity. Although residual Tergitol was not directly quantified in this study, extensive washing steps and the absence of cytotoxic or inflammatory effects suggest that residual detergent levels are low and compatible with basic cell viability. Systematic evaluation of detergent residuals and their impact on cardiac-specific recellularization will be addressed in future studies. Finally, matrix stability assays revealed slow degradation under physiologically relevant enzymatic conditions, suggesting preservation of ECM structural integrity over time. While this property is advantageous for maintaining scaffold mechanics, it may impose constraints on tissue remodeling and cell repopulation. Previous studies on decellularized cardiac tissues have reported faster degradation under higher enzyme concentrations, indicating that the slower kinetics observed here likely reflect both ECM composition and conservative enzymatic exposure (Nordsletten et al., 2021; Kimicata et al., 2020).

Collectively, these findings indicate that Tergitol-based decellularization enables the generation of an AVN-derived scaffold with preserved core ECM components, while maintaining mechanical behaviour and immunocompatibility within the experimental conditions evaluated. At the same time, microstructural organization, particularly in collagen orientation and network anisotropy, and selective depletion of regulatory ECM proteins represent potential limitations that may influence functional integration. Recognizing both the advantages and limitations of Tergitol versus more traditional SDS or Triton X-100 protocols is essential for interpreting scaffold translational potential and guiding future optimization strategies.

## 5 Conclusion

In this study, we demonstrated that Tergitol-based decellularization of porcine AVN generates scaffolds characterized by efficient cell removal, preservation of core ECM components, and minimal immunogenicity. Proteomic and ultrastructural analyses confirmed retention of major fibrillar collagens and proteoglycans, while mechanical testing indicated largely preserved elasticity, fatigue resistance, and viscoelastic behavior. SHG and 2D-FFT analyses revealed alterations in collagen fiber orientation, highlighting subtle microstructural changes that may influence mechanical performance and electrophysiological signaling.

These findings underscore the inherent trade-off in detergent-based decellularization: achieving thorough cell removal while minimizing ECM alterations. Overall, Tergitol-based decellularization represents a robust approach for generating AVN scaffolds with well-preserved structural, biochemical, and mechanical properties. Moreover, emerging approaches such as biologically inspired 3D printing could complement or substitute decellularized scaffolds, offering precise control over scaffold structure and cellular organization to enhance reproducibility and functional outcomes.

## Data Availability

The proteomic data presented in the study are deposited in the ProteomeXchange Consortium repository via the PRIDE, accession number PXD073301.
