# Supplementary material for: New frontiers in porcine atrioventricular node decellularization: preserving extracellular matrix architecture for biological scaffolds
Source: Front Bioeng Biotechnol. 2026 Mar 13;14:1766378. doi: 10.3389/fbioe.2026.1766378 (PMC13021660; doi:10.3389/fbioe.2026.1766378)
Supplement: Supplementary file 1 [file DataSheet1.pdf]

New frontiers in porcine atrioventricular node decellularization: preserving extracellular matrix architecture for biological scaffolds

Supplementary informations

Biocompatibility of Decellularized AVN Tissue

Immunocompatibility of decellularized AV tissue was first evaluated by assessing the presence of α-Gal epitopes. Analysis confirmed effective removal of α-Gal, indicating minimal potential for xenogeneic immune activation and supporting the scaffold’s suitability for translational applications. Subsequently, the potential of AV-derived tissue to influence capillary formation was assessed using an in vitro angiogenesis assay with endothelial cells. After 24 hours of incubation, cells exposed to conditioned medium derived from AV tissue exhibited capillary-like network formation comparable to that observed in the healthy control group. Quantitative analysis revealed no significant differences in key morphometric parameters, including the number of nodes, total tubule length, and number of segments, between the conditioned medium group and the healthy control. In contrast, treatment with DMSO resulted in a marked reduction in tubule formation, validating the assay’s sensitivity and specificity. Collectively, these results indicate that decellularized AV tissue effectively removes immunogenic α-Gal epitopes while the tissue secretome supports endothelial network formation in vitro (Supplementary Figure 1).

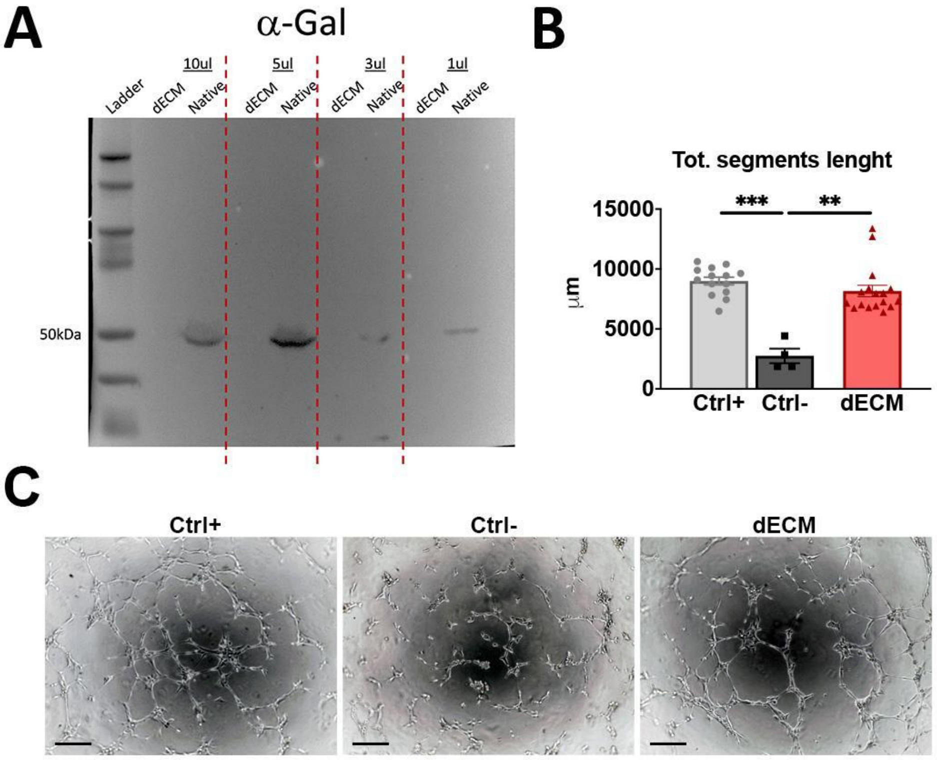

Supplementary Figure 1. Evaluation of Biocompatibility. (A) Western blot analysis at different protein concentrations confirming successful α-Gal removal in decellularized AVN tissue compared with native controls (n = 6 different pigs for native samples and n = 6 different pigs for decellularized samples). (B,C) Ctrl+: HUVEC grown with endothelial medium; Ctrl-: HUVEC grown with medium that inhibits growth; dECM: HUVEC grown with endothelial medium mixed with dECM; Tot. segments length: sum of length of the segments in the analyzed area.
